# Symphyseal Morphology in Sagittal Skeletal Discrepancies: A Retrospective Observational Study

**DOI:** 10.3390/dj13110544

**Published:** 2025-11-20

**Authors:** Francesca Squillace, Rosanna Guarnieri, Rachele Podda, Gabriella Galluccio, Roberto Di Giorgio, Ersilia Barbato

**Affiliations:** Department of Oral and Maxillofacial Sciences, School of Dentistry, Sapienza University of Rome, 00185 Rome, Italy

**Keywords:** mandibular symphysis, skeletal class, malocclusion, ANB angle, IMPA, cephalometric analysis, symphysis morphology, facial aesthetics

## Abstract

**Background:** The aim of this study was to evaluate the correlation between skeletal class and morphological patterns of the mandibular symphysis. **Methods:** The sample consisted of 90 patients with an average age of 18 years (44 > x > 12). In order to investigate any correlation between skeletal class and morphological patterns of the mandibular symphysis, the following tests were used: Pearson’s correlation test, Spearman’s test, and the analysis of variance test (ANOVA) followed by Tukey’s post hoc HDS test. The significance level was set at 0.050. **Results:** Pearson’s correlation test and ANOVA showed a weak negative correlation between malocclusion and symphyseal height. Therefore, as ANB increases, symphyseal height decreases (r = −0.25, *p* < 0.01). In addition, a strong positive correlation was found between ANB and IMPA (r = 0.47, *p* < 0.01). So, as ANB increases, IMPA increases. No statistically significant association was found between symphyseal morphology and IMPA (Spearman’s test). **Conclusions:** The analysis revealed a weak but statistically significant negative correlation between ANB angle and symphysis height, indicating that as sagittal discrepancy increases (higher ANB), the symphysis tends to be shorter (r = −0.25, *p* < 0.01). A moderate positive correlation was also found between ANB and IMPA (r = 0.47, *p* < 0.01), suggesting that a more pronounced Class II skeletal pattern is associated with greater incisor proclination. However, no significant relationship was observed between symphysis type (A/B/C) and IMPA. When comparing skeletal classes, Class III subjects displayed significantly greater symphyseal height than Class II subjects (*p* < 0.001), while Class II subjects showed the highest IMPA values (*p* < 0.001).

## 1. Background

The first definition of “normal occlusion” historically dates back to the late 19th century. It was enunciated by Edward H. Angle, who proposed his own classification of malocclusions based on the positional relationship of the upper permanent first molar to the lower [1].

Although Angle’s classification is still widely used in clinical practice today, it has the major limitation of referring to malocclusion exclusively in dentoalveolar terms. A few years later Ballard developed a classification of malocclusions based on the skeletal assessment of the sagittal relationship between the maxilla and mandible [2,3].

Finally, Ackermann and Proffit in the 1960s proposed a diagram that clarified the complexity of the interconnections in the three planes of space involved in defining a facial harmony (introducing the concept of aesthetics) [4].

Prevalence of malocclusion in different age groups ranges from 20% to 93% [5,6,7,8,9].

The chin symphysis is a specific type of cartilaginous joint, called the amphiarthrosis, the purpose of which is to connect the two hemimandibulae that are joined through the process of synostosis. The mandibular symphysis forms from the bilateral mandibular prominences of the first pharyngeal arch. Between the 4th and 5th weeks of development, these prominences enlarge and begin to fuse at the midline. From the 5th to 8th week, neural crest–derived ectomesenchyme gives rise to Meckel’s cartilage bilaterally, which acts as a template for the intramembranous ossification of the mandible. Fusion of the mandibular prominences results in the formation of the lower jaw, lower lip, and cheeks. The external midline fusion point is the mentum (chin). Incomplete mesenchymal fusion or ossification at this site may result in a midline cleft or dimple of the chin [10].

The symphysis joins at the age of 6–9 months and continues to grow until adolescence, undergoing retrograde and ascending growth changes with bone deposition on all surfaces except the region above the Pogonion, where bone resorption occurs [11].

Mandibular growth is most pronounced during childhood and the pubertal growth spurt; thereafter, residual but measurable changes persist into early adulthood, with substantial inter-individual variability in timing, duration and intensity. Longitudinal cohorts and implant studies consistently show that the adolescent peak is followed by smaller, ongoing changes rather than an abrupt cessation, and adult craniofacial changes are documented well beyond the late teens. Moreover, CBCT studies indicate completion of condylar cortical bone formation at approximately ~22 years in females and ~24 years in males, supporting the concept of residual mandibular maturation into the early twenties [12,13,14,15,16].

Symphysis morphology is characterized by significant individual variability, resulting from the interaction of several genetic, nongenetic, and adaptive factors [17].

The functional environment, according to some studies, influences the shape and size of the mandibular symphysis, a sign that the biomechanical loads of the masticatory cycle stimulate adaptive morphological responses. Other factors that may influence symphysis morphology and size are the vertical ratios of the mandible and the inclination of the lower incisors [18]. The morphology of the mandibular symphysis plays a key role in orthodontic diagnosis and treatment planning, as it is considered a primary reference point for the aesthetics of the facial profile and is decisive in determining the optimal positioning and long-term stability of the lower incisors during orthodontic treatment [14,19]. A mandibular symphysis with adequate bone volume and optimal angulation offers a stable structural base for positioning the lower incisors. In contrast, insufficient symphyseal support—characterized by thin bone, decreased height, or unfavorable inclination—may raise the risk of post-treatment relapse, tooth mobility, and periodontal problems like dehiscences or fenestrations. In addition, the symphysis is considered as one of the key factors in assessing the direction of rotation in mandibular growth.

In Ricketts’ evaluations, consistent with other studies in the literature, the morphology of the mandibular symphysis serves as a predictor of mandibular growth direction. Based on qualitative criteria, a thicker symphysis is associated with an anterior direction of growth.

A study of the literature has revealed that a thorough morphological assessment of the mandibular symphysis—considering individual sagittal and vertical skeletal growth patterns as well as genetic and environmental factors—is a valuable tool for proper orthodontic therapeutic planning [20]. Specifically, the mandibular symphysis has been categorized into three main morphological types—A, B, and C—based on differences in height, width, and angulation. Type A: tall and narrow symphysis with greater vertical height and reduced width, often associated with Class II skeletal patterns; type B: symphysis with moderate height and width, representing a balanced morphology typical of Class I; type C: shorter and wider symphysis with a more obtuse angle, commonly observed in Class III skeletal patterns [21].

Recent research has increasingly focused on the morphological variations in the mandibular symphysis and their clinical implications, highlighting their significance in understanding skeletal patterns, growth dynamics, and craniofacial anatomy to improve orthodontic diagnosis and treatment strategies.

Ruiz et al. investigated morphological changes in the mandibular symphysis during adolescence, highlighting the influence of vertical growth patterns on symphyseal form [22]. Another study investigated shape variations in the symphysis among Class III patients, underscoring the impact of gonial angle, incisor inclination, and sex on symphyseal form [23]. Furthermore, Ghafari et al. investigated variations in chin morphology and mandibular incisor length across different facial divergence patterns, providing new insights based on CBCT imaging [24].

The morphology of the mandibular symphysis (MS) may influence diagnosis and treatment planning in orthodontic patients. It could serve as an important anatomical reference point for facial aesthetics, particularly in the lower facial region. Predictive morphological evaluations might help guide orthodontic movements of the lower incisors, potentially reducing the risk of dehiscences and fenestrations, especially during dento-alveolar compensation therapies [20,21].

The purpose of the present study was to evaluate the possible correlation between subjects in Skeletal Class I, II and III and Symphyseal morphology. The starting null hypothesis is that there are no statistically significant differences in symphyseal morphology between Class I, II and III subjects.

## 2. Materials and Methods

Pretreatment records of 598 Caucasian patients treated at the Department of Orthodontics of “Sapienza” University of Rome were analyzed between January 2021 and May 2022. The original study was approved by the Department of Odontostomatological and Maxillofacial Sciences, Sapienza University of Rome (Authorization 56/2020, Protocol number 0001396; Approval date: 22 December 2020).

The following inclusion parameters were adopted for sample selection: age older than 12 years, permanent dentition, absence of agenesis, impacted, supernumerary and deciduous teeth, absence of gingivitis, complete medical records such as lateral cephalogram and plaster study models.

Given the well-documented variability in the timing and duration of adolescent craniofacial growth and the evidence for residual mandibular maturation into the early twenties, we retained subjects aged ≥12 years and treated age as a potential source of heterogeneity, explicitly acknowledging the associated limitations in the Discussion [12,14,15].

Exclusion parameters were incomplete medical records, craniofacial syndromes, systemic diseases, ongoing radiation or chemotherapy, history of head or neck surgery, lateral cephalograms not suitable for analysis, damaged plaster study models, and severe allergic conditions associated with chronic airway obstruction or craniofacial morphological alterations. A total of 90 subjects were selected from the original study sample.

The 90 patients were distributed as follows: 30 in Skeletal Class I; 30 in Skeletal Class II; and 30 in Skeletal Class III. The final male-to-female distribution within each skeletal class (approximately 1:1 ratio) occurred naturally as a result of the inclusion and exclusion criteria applied, with a mean age of 18 years. The equal distribution of skeletal classes in the sample was not intentional but reflects the composition of the available records at the time of data collection. This may not represent the general population, but no adjustments were made to avoid introducing selection bias.

**Figure 1:**
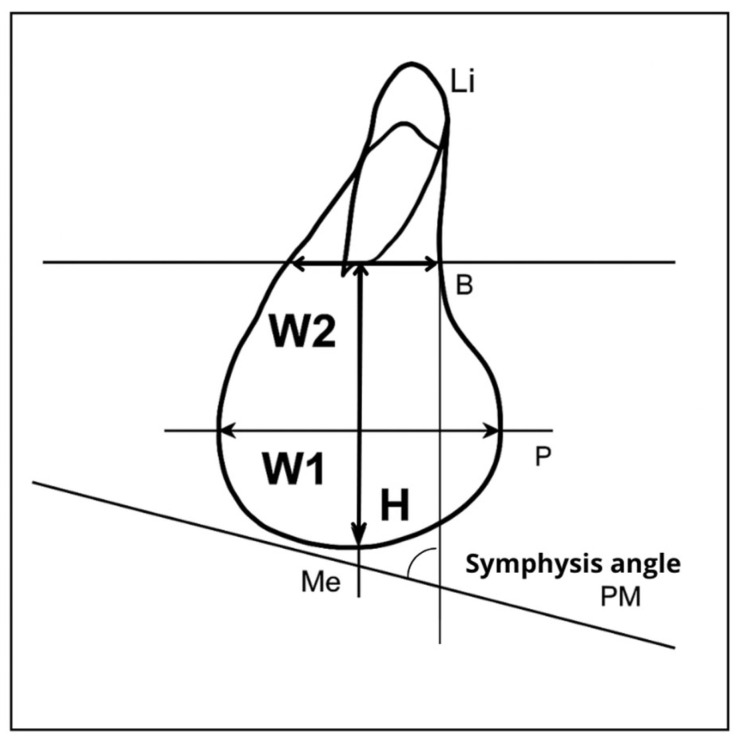
Graphical Representation of Angular and Linear Parameters of the mandibular symphysis.

The lateral cephalograms were digitalized using digital software (Product Name: Dental Imaging Software—Brand Name: WEBCEPH—Model Name: WEBCEPH—Software Version: 1.5.0—Date of Manufacture: 10 November 2020—FDA 510(k) Number: K220903; AssembleCircle Corporation, 2025; Seongnam-si, Gyeonggi-do, Republic of Korea) in order to obtain linear and angular skeletal measurements for morphological analysis of the mandibular symphysis (Figure 1).

### 2.1. Angular and Linear Parameters of the Mandibular Symphysis

Linear measurements:

1. *Height*: the perpendicular distance measured from the most caudal point of the lower margin of the symphysis to a reference line passing through point B.
2. *Width* 1: line joining the most anterior and posterior margin of the symphysis.
3. *Width* 2: line joining the most anterior and posterior (superior) margin of the symphysis, and passing through point B.

Angular measurements:

1. *Symphysis angle*: angle formed between the line passing through point B and the mandibular plane (pm, Go–Me).

### 2.2. Measurements Made on Digital Cephalometry

1. *SNA:* angle between the S point (sella turcica), the nasion (N) and the A point (supraspinal point of the maxilla);
2. *SNB:* angle between the S point (sella turcica), the nasion (N) and the B point (submental point of the jaw);
3. *ANB:* difference between the angular values SNA and SNB.

The measurements carried out on the digital cephalometry are reported in Table 1.

Following the cephalometric measurements obtained from the digital tracings, each mandibular symphysis was categorized into one of three morphological types—A, B, or C—according to its height, width, and angular characteristics. This classification is indicated in Table 1 under the column titled “TYPE.”

### 2.3. Statistical Analysis

Statistical analyses were conducted with the *STATA program* (version 17; developed by StataCorp LLC, College Station, TX, USA).

The sample size was determined using the formula:

$$n=\frac{N\;\times \;X}{X+N-1}$$

where

$$X=\frac{{Z^{2}}_{\alpha /22}\times p\times (1-p)}{{MOE}^{2}}$$

The sample size was established following a power analysis performed with the GPower program, which showed that the minimum number of subjects to be included in the analyses was 88 (Power = 0.80; *a* = 0.05; Effect size = 0.30).

A single operator (F.S.) came up with the sample selection criteria. The measurements were then entered into an Excel spreadsheet and statistically analyzed. To verify the reliability of the results that emerged from the first observation, all measurements were subjected to a second random evaluation by the same operator after a period of one month. The error between the two measurements was calculated using the Houston method [25].

The difference between the two observations in the analysis of skeletal measurements revealed a statistically nonsignificant error; a 98.7 percent equality rate was observed.

To test the research hypotheses, the following analyses were performed:

A. To investigate any correlations between ANB and symphysis morphology (symphysis width; symphysis height; symphysis angle; IMPA; symphysis type* A/B/C), the Pearson’s [26,27] correlation test was used.
B. To investigate possible correlations between symphyseal morphology [28] (type A/B/C → WIDTH2) and IMPA, Spearman’s test was used. The subdivision into symphysis types A, B, and C was made to allow the data to be statistically comparable. Following the criteria already described in the literature [23], patients were classified as follows: W2 < 7.3 mm → narrow symphysis: Group A; 7.3 ≤ W2 ≤ 8.3 mm → medium symphysis: Group B; W2 > 8.3 mm → wide symphysis: Group C.
C. To investigate possible correlations between malocclusion type (Skeletal Class I-II-III) and measurements of symphyseal morphology, the analysis of variance (ANOVA) test followed by the HSD post hoc Tukey test was used.

The significance level was set at 0.050.

## 3. Results

### 3.1. Sample Description

The sample consisted of 90 patients (30 in Skeletal Class I, 30 in Skeletal Class II, 30 in Skeletal Class III), with a mean age of 18 years (44 > x > 12). The sample was evenly distributed by sex, with a 1:1 ratio of males to females (M and F), as shown in Table 2.

### 3.2. Inferential Analysis

**Figure 2:**
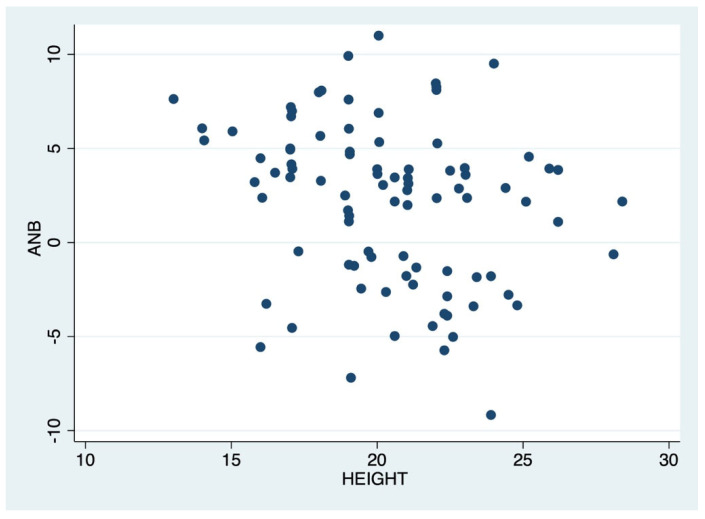
Graphical representation of the correlation between ANB and symphysis height.

A. Correlation between ANB values and symphysis morphology (symphysis width; symphysis height; symphysis angle; IMPA; symphysis type. Pearson’s correlation test [26,27] showed a weak negative correlation between malocclusion and symphyseal height. Therefore, as ANB increases, symphyseal height decreases (r = −0.25, *p* < 0.01) (Figure 2).

This scatterplot shows the relationship between symphyseal height (x-axis) and ANB angle (y-axis) in the sample. Each point represents one patient, illustrating variability in sagittal skeletal relationships and symphyseal height.

**Figure 3:**
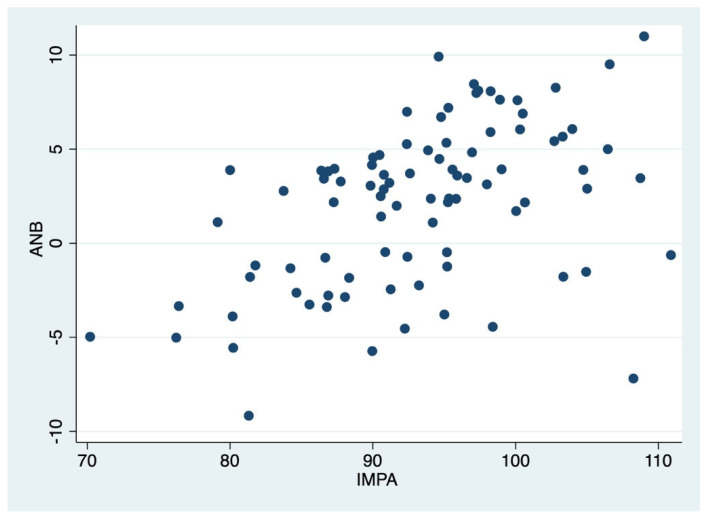
Graphical representation of the correlation between ANB and IMPA.

In addition, a strong positive correlation was found between ANB and IMPA (r = 0.47, *p* < 0.01). So, as ANB increases, IMPA increases (Figure 3).

This scatterplot shows the relationship between IMPA (x-axis) and ANB angle (y-axis) in the sample. Each dot represents a patient, illustrating the variability in sagittal skeletal relationships across different lower incisor inclinations.

B. Correlations between symphyseal morphology (CLASS A/B/C → Width2) and IMPA. No statistically significant association was found between symphyseal morphology and IMPA.
C. Correlations between malocclusion (I, II, III dento-skeletal class) and symphysis morphology (symphysis width; symphysis height; symphysis angle; IMPA; symphysis type A/B/C).

The results of the ANOVA test (Table 3) showed that symphysis height was significantly greater in Class III subjects in comparison to Class II subjects (*p* < 0.001). IMPA also proved to be highly significant, with a greater value in Class II subjects compared to those in Classes I and III (*p* < 0.001).

** *p* < 0.01; * *p* < 0.05. Abbreviations: SD, standard deviation.

## 4. Discussion

The morphology of the mandibular symphysis plays a crucial role in orthodontic diagnosis and treatment planning, serving as a key anatomical reference for facial aesthetics, particularly in the lower facial region. Understanding symphyseal shape and dimensions aids in determining safe limits for orthodontic movements of the lower incisors, thereby minimizing risks such as alveolar bone dehiscences and fenestrations. Incorporating these clinical implications enhances treatment precision and stability [29].

In addition, the MS has been considered as one of the predictors of the direction of mandibular growth rotation [30]. MS morphology is a complex phenotype that results from the interaction of several genetic, nongenetic and adaptive factors. Therefore, the purpose of the present study was to assess whether skeletal dimensional differences in symphysis morphology, among different skeletal classes, may be predictive of certain malocclusions and whether, when present, the careful assessment of these differences may provide diagnostic insights for treatment planning.

The starting null hypothesis is that there are no statistically significant differences in symphyseal skeletal morphology in Class I patients compared with Class II and Class III patients.

The study conducted analyzed the existence of a correlation between the morphological differences in the symphysis in the various sagittal discrepancies, in relation to ANB and IMPA value.

The morphology of the mandibular symphysis shows considerable individual variability, influenced by a combination of genetic, environmental, and functional factors. Elements such as vertical skeletal pattern, mandibular divergence, and lower incisor inclination play a key role in shaping the symphysis. In particular, vertical dimension appears to significantly affect symphyseal form in Class I and Class III subjects, with hypodivergent Class III individuals typically presenting a wider symphysis with reduced vertical height.

In addition, the symphysis is considered as one of the factors in assessing the direction of rotation in mandibular growth.

In our study, Pearson’s correlation test [26] showed a weak negative correlation between malocclusion and symphyseal height. Therefore, as ANB increases, symphyseal height decreases (r = −0.25, *p* < 0.01). The results of the ANOVA test confirmed the above, in fact, symphysis height was found to be greater, with a high level of significance, in Class III subjects in comparison to Class II subjects (*p* < 0.001). In contrast, the assessment of symphysis width in Class III subjects when compared with Skeletal Class I and Class II subjects was not significant. These data are in agreement with what is already found in the literature, for example, the study conducted by Jain S. et al. [20] evaluated the morphology of the mandibular symphysis and the angulation of the lower incisors in the different anteroposterior relationships of the skeletal bases and in the various skeletal growth patterns, with the aim of showing the existence of a correlation between morphology and dentofacial parameters. The observation was performed on a sample of 90 patients, divided into three groups—Class I, Class II and Class III—after assessing the angular parameter ANB. The results obtained showed that the total length of the mandibular symphysis was statistically greater in the Class III group than in the other two groups. In addition, there was an increase in the distance between the Pogonion and Menton in the Class III group compared to the Class I and Class II groups (Class III > Class I > Class II). However, the evaluation of symphysis width showed a lower value in Class III subjects. In Class III subjects, the authors observed a greater slope of the alveolar wall of the symphysis than in Class I and Class II subjects, concluding that the mandibular symphysis reflects compensation of the skeletal pattern of the mandible.

According to the outcome of the values obtained from the study conducted, IMPA was also shown to be highly significant, with a greater value in subjects in the second skeletal class compared to those in the first and third (*p* < 0.001). This finding is also consistent with previously reported results in the literature [20].

In 2017, Gomez Y. et al. [31] analyzed correlations between mandibular symphysis characteristics (height, prominence, tilt, concavity, and convexity) and facial patterns, skeletal class, mandibular incisor position, and sex. In addition, they investigated associations between symphysis soft tissue dimensions and underlying bony structures. Specifically, to evaluate the soft tissue thickness over the mandibular symphysis, linear distances were measured between corresponding skeletal and soft tissue landmarks. The following measurements were recorded (in millimeters): from the Pogonion (Pg) to the soft tissue Pogonion (Pg); from the bony Gnathion (Gn) to the soft tissue Gnathion (Gn); from the bony Menton (Me) to the soft tissue Menton (Me). The results of this study showed that mandibular symphysis soft tissue shape is only weakly correlated with the underlying skeletal structures. Furthermore, the authors of the study found that the mandibular symphysis has larger dimensions in men than in women, and that differences related to symphysis concavity between the sexes correlated with skeletal class. In line with what has been observed by other authors, the study of symphysis morphology does not show significant differences between skeletal classes and vertical patterns independently, but relationships are found when both parameters of class and vertical development are associated with each other.

Further morphological evaluation was performed by Linjawi AI. et al. [32] The authors analyzed, through linear and angular measurements, morphological variations in the mandibular symphysis in relation to gender and sagittal and vertical skeletal ratios in a sample of ethnic Saudis. A significant interaction between gender and sagittal skeletal ratio was found in the dento-alveolar length, length and vertical dimension of the mandibular symphysis. Only the parameter related to dento-alveolar symphysis length was significant in the comparative evaluation between sex and vertical skeletal ratio.

Sadek et al. [33] in their studies analyzed alveolar bone thickness, symphysis shape and facial type (normofacial, brachyfacial and dolichofacial) and found some correlations. Evaluations showed that the dolichofacial type with accentuated mandibular plane is characterized by a narrower symphysis and less anterior element support alveolar bone than subjects with brachyfacial type and flat mandibular plane. The hypothesis that a narrow symphysis morphology is related to thin alveolar bone and a wide morphology is associated with thick alveolus appears to be validated by numerous studies. In contrast, mandibular morphology does not appear to be a determining factor in the development of gingival recessions.

In addition, the vertical hypodivergence relationship also affects the bony thickness of the symphysis. Brachyfacial subjects have greater symphysis bone thickness than dolichofacial subjects, which provides greater structural support for the mandibular incisors, allowing for more dynamic dentoalveolar correction of sagittal skeletal discrepancies [34].

The thickness of the bone present posterior to the apex of the mandibular incisors appears to be increased in Class III subjects compared to the bony counterpart present anteriorly. Therefore, the root apex appears to be closer to the labial cortical of the symphysis. Based on the results obtained from the studies, a proper pretreatment assessment of the angulation of the lower incisors and their proximity relationships with the symphysis alveolar bone is necessary to properly plan the limits of orthodontic movement of the incisors in order to avoid root resorption or dehiscence of the associated tissues [35].

Aki, Todd et al. [11] studied the mandibular symphysis by measuring various structural features, particularly symphysis height and width. In the observation conducted by the Authors, it was possible to understand how in a wider symphysis the protrusion of the incisors is aesthetically acceptable and allows greater possibility of performing nonextractive orthodontic treatment. Conversely, in a symphysis with a greater increase in vertical dimension and incisor protrusion, an extractive orthodontic treatment is more commonly indicated. Finally, the assessment of the bone density of the mandibular symphysis conducted by Authors Gousman J. et al. [36], through a retrospective study with analysis by Cone-Beam Computer Tomography (CBCT), allowed us to observe how vertical and horizontal skeletal patterns influence, in addition to the symphysis morphology, the density of the bone tissue itself. In hyperdivergent skeletal patterns, bone cortical density appears to be statistically higher than that observed in normodivergent and hypodivergent skeletal patterns at the Menton (ME) level. Normodivergence is in turn associated with higher bone density than the hypodivergent skeletal pattern. Similarly, the same study on Class II skeletal bases showed a significant increase in symphyseal bone density compared with Class I and Class III sagittal skeletal ratios. Ref. [36] Higher bone density offers control over orthodontic movements of the lower incisors, reducing the risk of developing dehiscences and fenestrations during dento-alveolar compensation therapies. In this regard, as reported by the work of Al-Khateeb SN. et al. [21], variation in mandibular incisor inclination in sagittal skeletal discrepancy compensations could cause remodeling of the mandibular symphysis surface, significantly affecting its morphology [37].

This study presents valuable insights into the morphological variations in the mandibular symphysis across different sagittal skeletal classes; however, several limitations should be acknowledged. Notably, vertical facial patterns (e.g., brachyfacial, dolichofacial) were not evaluated, despite their known influence on craniofacial growth and symphyseal shape. Our cross-sectional, retrospective design cannot resolve longitudinal growth trajectories in individuals, and including participants who may still undergo residual mandibular changes introduces unavoidable confounding. Nonetheless, this choice preserves statistical power and representativeness, given the large inter-individual variability in the timing/cessation of adolescent growth and documented mandibular maturation into the early twenties [12,13,14,15]. Accordingly, results are interpreted as associations rather than causal effects, and conclusions are tempered to reflect this limitation. Additionally, the homogeneity of the sample in terms of ethnicity limits the generalizability of the findings, as ethnic differences have been shown to affect craniofacial structures. Future research should aim to include a more diverse population and incorporate vertical skeletal parameters to provide a more comprehensive understanding of the factors influencing symphyseal morphology. Although patients aged 12 and above were included to capture early morphological features relevant to orthodontic planning, we acknowledge that mandibular growth continues beyond this age, potentially altering skeletal classification. This limitation introduces variability in our findings and highlights the need for longitudinal studies to assess morphological changes over time. From a clinical perspective, understanding these morphological patterns may help optimize orthodontic treatment planning, particularly in cases where incisor positioning is limited by the bony envelope.

## 5. Conclusions

The statistical analysis of the data obtained from the study sample revealed the following findings:

1. Symphysis height is higher in Class III subjects compared to Class II subjects, with a high level of statistical significance (*p* < 0.001). Therefore, as ANB increases, symphysis height decreases (r = −0.25, *p* < 0.01).
2. The assessment of symphysis width in Class III subjects showed no statistically significant difference when compared to Class I and Class II skeletal subjects.
3. The evaluation of the symphysis angle in Class III subjects revealed no statistically significant difference when compared to Class I and II skeletal subjects.

These findings suggest that vertical dimensions of the symphysis, particularly height, may play a more critical role in characterizing skeletal discrepancies than width or angular measures. Importantly, the early assessment of symphysis morphology—especially in Class III patients—could provide valuable insight into growth trends and support more individualized orthodontic treatment planning. Integrating such morphological indicators into routine diagnostic assessments may enhance the precision and timing of interventions, leading to more effective treatment outcomes.

## Figures and Tables

**Table 1:** Measurements carried out on digital cephalometry.

ID	SC	Sex	Age	ANB	Height	Width 1	Width 2	Type	SA°	IMPA
Patient 1	1	0	13	2.37	23.08	16.04	6.10	A	62	94.06
Patient 2	1	0	15	2.78	21.03	14.05	6.30	A	69	83.75
Patient 3	1	0	17	3.28	18.07	14.03	7.70	B	66	87.75
Patient 4	1	0	16	3.89	21.08	10.06	7.80	B	53	80.00
Patient 5	1	0	16	2.38	16.06	13.40	7.90	B	64	95.33
Patient 6	1	1	19	3.21	15.8	14.20	8.80	C	75	91.16
Patient 7	1	1	14	1.71	19	13.02	8.40	C	62	100.04
Patient 8	1	0	14	3.71	16.5	13.10	6.50	A	57	92.60
Patient 9	1	1	15	3.47	17.02	14.40	5.40	A	60	96.59
Patient 10	1	0	17	2.18	28.40	16.70	6.30	A	62	87.26
Patient 11	1	1	22	1.42	19.04	15.02	7.10	A	80	90.58
Patient 12	1	1	22	3.82	22.5	10.05	5.90	A	60	86.87
Patient 13	1	1	16	2.36	22.04	17.01	7.40	B	65	95.83
Patient 14	1	1	13	3.64	20.01	14.06	8.20	B	66	90.78
Patient 15	1	1	14	1.1	26.2	14.07	7.00	A	66	94.20
Patient 16	1	0	16	3.43	21.05	12.02	6.40	A	55	86.57
Patient 17	1	1	14	3.96	23	10.09	5.40	A	57	87.31
Patient 18	1	0	16	2.18	20.60	17.90	6.40	A	66	95.26
Patient 19	1	1	17	2.5	18.90	10.90	7.20	A	69	90.55
Patient 20	1	1	13	1.99	21.04	16.06	5.90	A	70	91.67
Patient 21	1	0	15	2.87	22.80	15.20	6.90	A	67	90.77
Patient 22	1	0	15	2.90	24.40	12.70	6.50	A	67	105.01
Patient 23	1	0	16	1.12	19.03	11.80	6.50	A	55	79.13
Patient 24	1	1	13	3.06	20.2	14.03	6.40	A	58	89.84
Patient 25	1	0	12	3.13	21.07	15.01	6.30	A	73	97.99
Patient 26	1	1	13	3.46	20.60	14.30	7.80	B	54	108.74
Patient 27	1	1	12	3.93	25.9	14.01	6.70	A	61	99.02
Patient 28	1	1	14	3.60	23.03	13.00	6.30	A	61	95.91
Patient 29	1	1	15	2.17	25.1	16.04	5.80	A	62	100.65
Patient 30	1	1	15	3.86	26.2	12.06	5.70	A	55	86.39
Patient 31	2	1	44	9.51	24.00	10.04	5.30	A	54	106.59
Patient 32	2	0	18	4.69	19.06	14.04	5.60	A	62	90.47
Patient 33	2	0	19	6.05	19.03	11.09	6.50	A	60	100.31
Patient 34	2	0	12	3.90	20.00	14.09	8.30	C	65	104.74
Patient 35	2	1	13	7.60	19.02	15.09	7.80	B	68	100.13
Patient 36	2	0	12	5.91	15.04	12.06	8.00	B	65	98.24
Patient 37	2	0	12	4.48	16.00	13.04	6.70	A	80	94.66
Patient 38	2	0	13	5.00	17.02	16.08	8.40	C	62	106.46
Patient 39	2	1	21	9.92	19.01	13.05	5.40	A	62	94.61
Patient 40	2	1	16	3.92	17.09	13.01	6.70	A	62	95.58
Patient 41	2	0	16	5.67	18.05	14.03	7.90	B	66	103.30
Patient 42	2	1	23	8.27	22.03	13.09	5.70	A	52	102.81
Patient 43	2	1	12	6.07	14.00	10.09	7.00	A	61	103.97
Patient 44	2	1	16	8.08	18.09	14.08	6.70	A	71	98.25
Patient 45	2	0	12	6.99	17.08	13.03	6.10	A	60	92.39
Patient 46	2	0	16	5.34	20.07	15.06	7.80	B	59	95.15
Patient 47	2	1	21	11.00	20.05	12.09	6.70	A	59	109
Patient 48	2	1	16	5.27	22.06	11.07	7.20	A	60	92.38
Patient 49	2	0	17	4.94	17.02	13.01	8.00	B	60	93.87
Patient 50	2	0	29	7.99	18.00	15.02	9.60	C	68	97.25
Patient 51	2	1	22	7.63	13.02	11.02	8.50	C	63	98.90
Patient 52	2	1	15	4.83	19.06	12.09	7.30	B	60	96.95
Patient 53	2	0	18	5.43	14.07	12.07	9.30	C	62	102.71
Patient 54	2	1	37	7.20	17.04	14.01	6.70	A	64	95.29
Patient 55	2	0	14	8.11	22.03	17.00	11.40	C	74	97.39
Patient 56	2	1	15	8.46	22.01	12.03	6.50	A	51	97.08
Patient 57	2	1	15	6.71	17.05	11.05	8.10	B	70	94.78
Patient 58	2	0	14	4.56	25.2	13.06	6.00	A	58	90.02
Patient 59	2	0	14	6.89	20.05	15.08	8.60	C	76	100.50
Patient 60	2	1	13	4.16	17.06	11.00	6.20	A	69	89.94
Patient 61	3	0	18	−4.54	17.08	19.09	5.60	A	50	92.24
Patient 62	3	1	20	−2.24	21.23	12.23	5.50	A	58	93.23
Patient 63	3	0	19	−1.78	21.00	10.00	9.60	C	64	103.34
Patient 64	3	1	20	−1.18	19.03	13.80	9.40	C	65	81.77
Patient 65	3	0	19	−2.86	22.40	13.30	7.20	A	65	88.05
Patient 66	3	0	21	−3.89	22.40	12.10	6.30	A	62	80.18
Patient 67	3	0	19	−2.63	20.30	13.60	6.90	A	56	84.65
Patient 68	3	1	26	−3.39	23.30	12.50	8.20	B	59	86.78
Patient 69	3	0	20	−9.17	23.90	11.40	5.70	A	61	81.31
Patient 70	3	0	19	−5.02	22.60	15.30	9.80	C	61	76.23
Patient 71	3	0	23	−4.97	20.60	11.00	7.00	A	61	70.20
Patient 72	3	1	24	−7.19	19.10	11.40	5.80	A	67	108.25
Patient 73	3	0	21	−5.56	16.00	11.70	6.50	A	62	80.22
Patient 74	3	0	26	−0.77	19.80	10.40	5.70	A	57	86.67
Patient 75	3	0	21	−1.33	21.34	12.43	7.30	B	62	84.23
Patient 76	3	1	24	−3.26	16.20	10.30	5.40	A	65	85.56
Patient 77	3	1	21	−0.48	19.70	12.30	8.50	C	67	95.19
Patient 78	3	1	19	−1.24	19.21	15.23	7.80	B	61	95.21
Patient 79	3	1	24	−4.44	21.90	11.90	6.40	A	71	98.40
Patient 80	3	1	24	−0.47	17.30	11.20	8.00	B	57	90.87
Patient 81	3	1	18	−5.73	22.30	15.70	8.40	C	54	89.96
Patient 82	3	0	22	−0.63	28.1	15.70	8.90	C	67	110.88
Patient 83	3	1	19	−1.52	22.40	12.70	6.00	A	68	104.94
Patient 84	3	1	18	−0.72	20.9	13.10	6.90	A	59	92.42
Patient 85	3	1	24	−3.79	22.30	10.90	4.50	A	54	95.01
Patient 86	3	1	23	−1.79	23.90	12.30	6.30	A	57	81.40
Patient 87	3	0	19	−2.78	24.50	12.40	8.20	B	67	86.88
Patient 88	3	0	18	−3.34	24.80	13.90	6.50	A	50	76.41
Patient 89	3	0	25	−2.45	19.45	15.21	7.20	A	53	91.25
Patient 90	3	1	21	−1.84	23.41	13.22	6.40	A	51	88.34

**Table 2:** Distribution of the sample by gender.

	N°	%M	%F
SAMPLE	90	48	52
CLASS I	30	30	36
CLASS II	30	35	32
CLASS III	30	35	32

**Table 3:** Morphological Parameters of the Mandibular Symphysis.

Parameters	Class I (n = 30)		Class II (n = 30)		Class III (n = 30)		ANOVA *p*-Value	Tukey’s Post Hoc HSD Test		
	Mean	±SD	Media	±SD	Mean	±SD		I and II *p*-Value	I and III *p*-Value	II and III *p*-Value
Height	21.3	3.2	18.6	2.9	21.2	2.7	0.001 **	0.002 **	0.994	0.002 **
Width1	13.8	2.1	13.1	1.7	12.9	1.9	0.136	0.255	0.152	0.956
Width2	6.8	0.9	7.3	1.4	7.1	1.3	0.209	0.180	0.620	0.673
α symphysis	63.2	6.5	63.4	6.6	60.4	5.7	0.111	0.992	0.184	0.145
IMPA	92.4	6.7	98.1	5.1	89.3	9.5	0.001 **	0.009 **	0.246	0.001

** *p* < 0.01; * *p* < 0.05. Abbreviations: SD, standard deviation.

## Data Availability

The original contributions presented in this study are included in the article. Further inquiries can be directed to the corresponding author.
